# Evaluation of Disocclusion During Protrusive and Laterotrusive Movements

**DOI:** 10.7759/cureus.32306

**Published:** 2022-12-07

**Authors:** Roshni Kumar, Vineet Sharma, Rahul Madaan, Balwant S Gurjar, Lisa Debbarma, Monika Saini

**Affiliations:** 1 Prosthodontics, RUHS College of Dental Sciences, Jaipur, IND; 2 Dentistry, RUHS College of Medical Sciences, Jaipur, IND

**Keywords:** disocclusion, stomatognathic system, indirect record, direct record, occlusion

## Abstract

Introduction

The importance of disocclusion in maintaining the health of the stomatognathic system is well established. However, the quantification of the same is limited. This study aimed to determine the amount of posterior teeth disocclusion in protrusive and laterotrusive movements to establish the desired disocclusion in occlusal treatments.

Materials and methods

Twenty dentate subjects with Angles Class I occlusion, 18-30 years, were included in the study. Direct and indirect techniques measured disocclusion at the mesiobuccal cusp tip of the mandibular first molar. In the direct technique, the protrusive and working and nonworking records were made intraorally at the edge-to-edge position of maxillary and mandibular central incisors and canines, respectively. For the indirect technique, putty indices were made on a semi-adjustable articulator at 2 millimeters (mm) eccentric movements and the disocclusion records were then made intraorally using indices at the predetermined excursions. The records were trimmed, and the disocclusion was measured using an optical microscope (ZEISS Axio Imager 2; Carl Zeiss Microscopy Deutschland GmbH, Oberkochen, Germany). The comparison of disocclusion by both techniques was done by paired t-test. The Pearson correlation coefficient was used to analyze the statistical correlation between the disocclusion obtained during different excursive movements.

Results

The mean disocclusion obtained by direct technique was 1.72 ± .49 mm in protrusion, 1.19 ± .50 mm for the working side, and 2.54 ± .70 mm for the nonworking side. For the indirect technique, the disocclusion obtained was 1.22 ± .37 mm in protrusion, 8.57 ± .33 mm for the working side, and 1.71 ± .61 mm for the nonworking side. On comparison, there was a statistically significant difference (*p*<0.05) seen for the values between the groups for direct and indirect subgroups except for the left working subgroup (*p*>0.05) with higher values in the direct group.

Conclusions

The disocclusion obtained by the direct technique was higher than that obtained by the indirect technique. For both techniques, as the working side disocclusion increased, the nonworking side disocclusion also increased.

## Introduction

Occlusion is the static relationship between the incising or masticating surfaces of the maxillary or mandibular teeth or tooth analogs [1]. Interocclusal contact is also one of the main features of occlusion. Interocclusal contact should be distributed on all tooth surfaces. This helps in distributing chewing forces in a proper manner concerning the long axes of the teeth [2]. For optimum occlusion, teeth harmonize with muscles, bones, ligaments, and nerves [3]. Therefore, occlusal rehabilitation should permit the efficient functioning of the stomatognathic system.

In the early years, investigators advocated balanced occlusion in natural dentition, a concept proposed by Bonwill. Monson amalgamated the principles of bilateral balanced occlusion, Bonwill's 4-inch triangle, Von Spee's compensating curve, and Balkwill and Christensen's observations on condylar movements to develop a three-dimensional occlusal philosophy. This occlusal model was known as the "spherical theory" and was one of the first attempts to present a working theory of three-dimensional occlusal concepts [4].

McCollum and Stuart [5] introduced the gnathological concept in their "research report." The principles of mandibular movements, transverse hinge axis, maxillomandibular relationships, and a fully adjustable arcon articulator were developed due to their observations. However, even the gnathological advocates supported the concept of balanced occlusion to restore natural dentition.

Schuyler [6] and Stuart [7] observed clinical failures in natural dentition restored with bilateral, simultaneous occlusal contact of the anterior and posterior teeth in excursive movements. Balancing contacts were identified as a common contributor to the loss of alveolar support of the posterior teeth and temporomandibular joint disorder. This led to the introduction of group function theory by Clyde and Schuyler in 1959 [8].

Simultaneously, D'amico [9], in his anthropological study on the skulls of primitive men and Native Americans, found excessive abrasion in the dentitions. In contrast, no abrasion was observed in the dentition of anthropoids with large cuspids, which discluded maxillary and mandibular cusps during eccentric movement. This was the inception of canine-guided occlusion, a cuspid-protected articulation that was a natural adaptation for preventing destructive occlusion. The concept was modified to mutually protected articulation, an occlusal scheme in which the posterior teeth prevent excessive contacts of the anterior teeth in maximum intercuspation and the anterior teeth prevent the posterior teeth in excursive movement [10].

Disocclusion is commonly described as the separation of opposing posterior teeth during eccentric movements of the mandible. Mohan et al. [11], Williamson et al. [12], and Manns et al. [13] provided irrefutable evidence of the role of disocclusion in reducing muscle activity.

Dawson [14] emphasized the role of disocclusion irrespective of the amount (of disocclusion) in optimizing occlusion. Scott et al. and Sooshan et al. [15,16] proposed a minimum of 0.5 millimeters (mm) disocclusion of molars on the nonworking side. Still, Hobo and Takayama [17-22] found disocclusion during protrusive movement and on the nonworking and working sides during lateral movements averaging 1.06 mm, 1.00 mm, and 0.47 mm, respectively.

The disocclusion of the posteriors should compensate for cuspal height, condylar path, and anterior influence to have a physiologically and orthopedically harmonious occlusion [3,14,17]. This study aims to determine the amount of posterior teeth disocclusion in protrusive and laterotrusive movements using direct and indirect (predetermined indexed) techniques to establish the desired disocclusion in occlusal treatments.

## Materials and methods

Before the study, the approval of the institutional ethical committee of RUHS College of Dental Sciences (RUHS-CDS/EC/2018/Proposal/0014) and informed consent of each participant was obtained. The participant data were formulated and used for research purposes.

Study design and setting

This in-vitro cross-sectional study was conducted in the department of prosthodontics, RUHS College of Dental Sciences, Jaipur, India.

Study participants

Twenty subjects in the age range of 18-30 years with Angle's class I malocclusion, the full complement of teeth till the second molar, no morphological or temporomandibular joint (TMJ) pathology, and no history of any dental intervention were selected irrespective of their sex.

**Figure 1:**
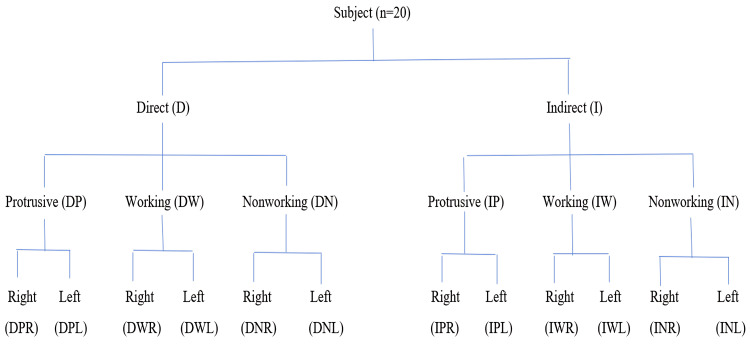
Flow chart: Interocclusal records n= Number, D= Direct, I= Indirect, DP= Direct Protrusive, DW= Direct Working, DN= Direct Nonworking, IP= Indirect Protrusive, IW= Indirect Working, IN= Indirect Nonworking, DPR= Direct Protrusive Right, IPR= Indirect Protrusive Right, DPL= Direct Protrusive Left, IPL= Indirect Protrusive Left, DWR= Direct Working Right, IWR= Indirect Working Right, DNR= Direct Nonworking Right, INR= Indirect Nonworking Right, DWL= Direct Working Left, IWL= Indirect Working Left, DNL= Direct Nonworking Left, INL= Indirect Nonworking Left.

An irreversible hydrocolloid impression (Zelgan; Dentsply, New Delhi, India) of maxillary and mandibular arches was made for each subject during the first appointment. The impressions were disinfected and poured into a Type III dental stone (Kalstone; Kalabhai Karson Private Limited, Mumbai, India). A facebow record was taken to orient the maxillary cast on a semi-adjustable articulator (Hanau™ Wide-Vue; WhipMix, Louisville, KY, USA). Direct and indirect techniques were used to quantify the disocclusion at the mesiobuccal cusp tip of the mandibular first molar during protrusive and laterotrusive movements (Figure 1).

**Figure 2:**
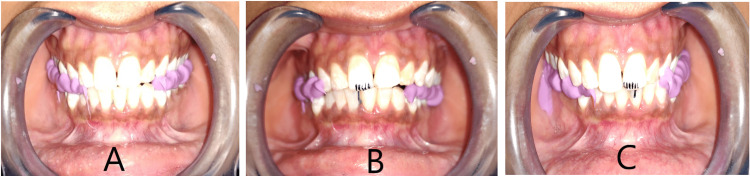
Direct interocclusal records (A) Protrusive record (B) Right lateral record (C) Left lateral record

The direct technique required training of the subjects to perform desired movements. Direct protrusive records required protrusion to achieve the edge-to-edge position of the maxillary and mandibular central incisors. At this position, silicone bite registration material (Occlufast Rock; Zhermack SpA, Badia Polesine, Italy) was injected to obtain intraoral direct protrusive right (DPR) and direct protrusive left (DPL) records (Figure 2). To standardize the amount of lateral movement in the frontal plane, lines were marked intraorally with a marker pen on the maxillary central incisor at 1 mm intervals. A line corresponding to the maxillary midline was marked on the mandibular central incisor in the centric occlusion position. Subjects were then given a face mirror and trained to move the mandible laterally. Disocclusion was measured when the last tooth contact occurred on maxillary and mandibular canines in canine-guided and canine and premolars in group function occlusion [23]. The interocclusal records were then made at this position. The procedure was performed for both left and right lateral movements, both for working and nonworking sides, to obtain intraoral direct working right (DWR), direct nonworking left (DNL), direct working left (DWL), and direct nonworking right (DNR) records.

**Figure 3:**
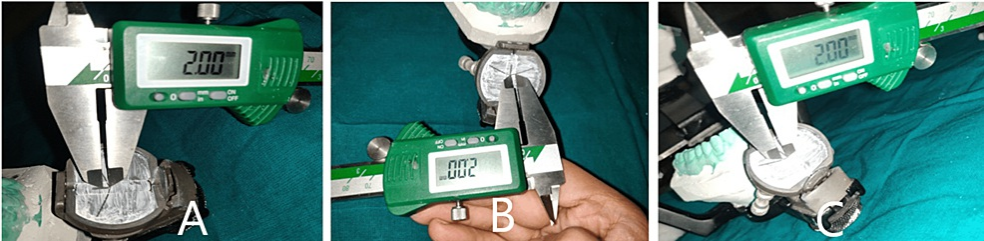
2 mm Mark on (A) Protrusive, (B) Right and (C) Left lateral pathway

**Figure 4:**
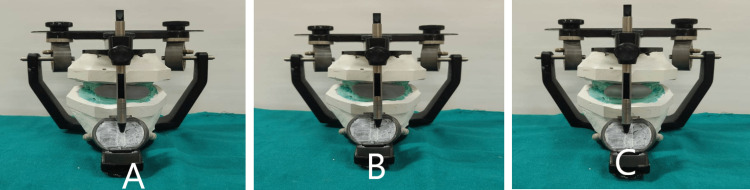
Indirect silicone index (A) Protrusive index (B) Right lateral index (C) Left lateral index

The protrusive records obtained in the intraoral direct technique were used to program the articulator. The horizontal condylar guidance was determined using this record, and the Bennett angle was calculated using Hanau's formula. The anterior guide table was programmed using the protrusive and laterotrusive records. The incisal pin was then inverted with the spherical tip towards the anterior guide table. The index for the indirect technique was made with condensation silicone putty (Zetaplus; Zhermack SpA) using the anterior teeth as a guide following 2 mm movement of the incisal pin on the protrusive and laterotrusive pathways and with centric locks in position. The index was then placed in the subject's mouth, and the subject was trained to close the mandible at this predetermined position (Figures 3, 4).

**Figure 5:**
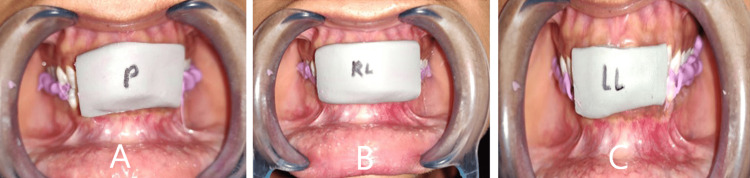
Indirect interocclusal records (A) Protrusive record (B) Right lateral record (C) Left lateral record

The protrusive record was made using the extraoral protrusive silicone index. The mandible was closed at this predetermined position. Silicone bite registration material was injected on both the right and left sides to obtain indirect protrusive right (IPR) and indirect protrusive left (IPL) records. The incisal pin was moved 2 mm on the left laterotrusive path, and the centric locks were tightened. Thus, the index was used to guide the left laterotrusive movements for the indirect working left (IWL) and indirect nonworking right (INR) records. The procedure was repeated for right lateral movements for indirect working right (IWL) and indirect nonworking left (INL) (Figure 5).

**Figure 6:**
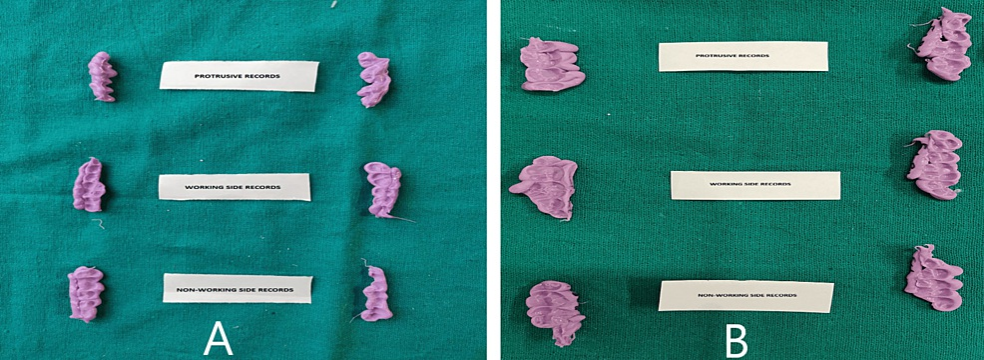
Interocclusal records (A) Direct technique (B) Indirect technique

**Figure 7:**
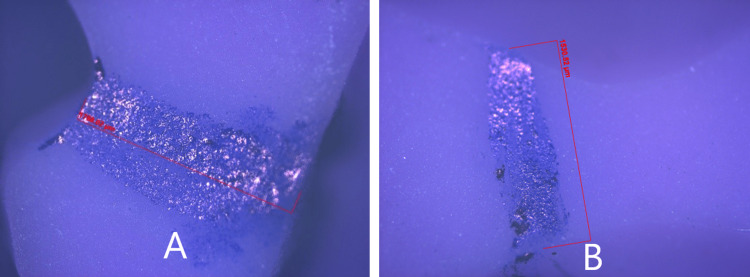
Digital image of silicone interocclusal record under 100x optical microscope (A) Direct interocclusal record (B) Indirect interocclusal record

The records obtained were disinfected in 2% glutaraldehyde for 10 minutes. Twelve records were obtained from each subject (Figure 6). The records were verified on the cast for complete seating. The mesiobuccal cusp tip of the mandibular first molar was marked using a marker pen. The index was cut mesiodistally using a Bard Parkers blade no.23 at this position. The records were trimmed buccolingually to obtain 2 cm in width samples for viewing and measuring under the optical microscope (ZEISS Axio Imager 2; Carl Zeiss Microscopy Deutschland GmbH, Oberkochen, Germany) (Figure 7).

Statistical analysis

The comparison of disclusion by both techniques was done by paired t-test. The Pearson correlation coefficient was used to analyze the statistical correlation between the disclusion obtained during different excursive movements.

## Results

The amount of disocclusion was evaluated at the mesiobuccal cusp tip of the mandibular first molar by two techniques, i.e., direct and indirect techniques in protrusive and laterotrusive mandibular movements under 5x magnification of the optical microscope. The direct technique showed a disocclusion of 1.73 ± .48 mm, 1.72 ± .52 mm, 1.18 ± .42 mm, 1.19 ± .57 mm, 2.52 ± .66 mm, and 2.57 ± .74 mm for DPR, DPL, DWR, DWL, DNR, and DNL respectively.

The indirect technique showed a disocclusion of 1.15 ± .33 mm, 1.28 ± .41, 0.82 μm ± 0.30 mm, 0.90 ± .36 mm, 1.69 ± .63 mm and 1.72 ± 0.60 mm for IPR, IPL, IWR, IWL, INR, and INL respectively. The statistical comparison showed a significant difference (p<0.05) between direct and indirect groups except for left lateral working (p>0.05) (Table 1).

**Table 1:** Comparison of Disocclusion obtained by Direct and Indirect techniques DPR= Direct Protrusive Right, IPR= Indirect Protrusive Right, DPL= Direct Protrusive Left, IPL= Indirect Protrusive Left, DWR= Direct Working Right, IWR= Indirect Working Right, DNR= Direct Nonworking Right, INR= Indirect Nonworking Right, DWL= Direct Working Left, IWL= Indirect Working Left, DNL= Direct Nonworking Left, INL= Indirect Nonworking Left, **= Highly Significant, *= Significant, #= Non Significant

Group	Mean	Standard Deviation	Standard Error of Mean	P value
DPR	1.73 mm	.48 mm	1.06 mm	.001**
IPR	1.15 mm	.33 mm	.07 mm	
DPL	1.72 mm	.52 mm	1.17 mm	.006**
IPL	1.28 mm	.41 mm	.09 mm	
DWR	1.18 mm	.42 mm	.09 mm	.003**
IWR	.82 mm	.30 mm	.07 mm	
DNR	2.52 mm	.66 mm	.15 mm	.001**
INR	1.69 mm	.63 mm	.14 mm	
DWL	1.19 mm	.57 mm	.13 mm	.057#
IWL	.90 mm	.36 mm	.08 mm	
DNL	2.57 mm	.74 mm	.17 mm	.001**
INL	1.72 mm	.60 mm	.13 mm	

On correlating the disocclusion within the direct and indirect groups, there was a statistically significant, positive but low correlation between (p<0.05) direct nonworking and direct protrusive subgroups, indirect nonworking and indirect working subgroups, and direct nonworking and direct working subgroups (Table 2). A positive correlation indicates that as the value of one variable increases, the other also increases. The mean disocclusion obtained for DP, IP, DW, IW, DN, and IN were 1.72 ± .49 mm, 1.22 ± .37 mm, 1.19 ± .50 mm, .86 ± .33 mm, 2.54 ± .70 mm, and 1.71 ± .61 mm respectively (Table 3).

**Table 2:** Correlation amongst subgroups within the Direct and Indirect groups DW= Direct Working, DP= Direct Protrusive, DN= Direct Nonworking, IW= Indirect Working, IP= Indirect Protrusive, IN= Indirect Nonworking, *= Significant, **= Highly Significant

Groups	DW and DP	DN and DP	DN and DW	IW and IP	IN and IP	IN and IW
Pearson Correlation value (R Value)	.239	.395*	.407**	.008	.166	.325*
P Value	.138	.012	.009	.962	.305	.041
Number	40	40	40	40	40	40

**Table 3:** Mean of obtained data DP= Direct Protrusive, DW= Direct Working, DN= Direct Nonworking, IP= Indirect Protrusive, IW= Indirect Working, IN= Indirect Nonworking

Group	Number	Minimum	Maximum	Mean	Standard Deviation
DP	40	.76 mm	3.10 mm	1.72 mm	.49 mm
DW	40	.17 mm	2.32 mm	1.19 mm	.50 mm
DN	40	1.24 mm	4.61 mm	2.54 mm	.70 mm
IP	40	.52 mm	2.32 mm	1.22 mm	.37 mm
IW	40	.34 mm	2.03 mm	.86 mm	.33 mm
IN	40	.59 mm	3.30 mm	1.71 mm	.61 mm

## Discussion

Extensive studies to establish the ideal occlusal scheme to be followed for the complete rehabilitation of the stomatognathic system led to various concepts in occlusion. Early proponents of balanced occlusion observed failures caused by unequal wear of the buccal and lingual cusps. This resulted in deflective occlusal contacts or interferences with a loss of centric-related closure. Also, the loss of alveolar support around posterior teeth in patients restored with balanced occlusion was observed [4,6,8].

Mutually protected occlusion (organic occlusion) evolved following research by Stuart et al. [10]. Organic occlusion encompasses disocclusion, cusp-to-fossae relationship, centric occlusion, uniform centric contact, forces directed in line with the long axes of the teeth, tripods, twin centric contact for cross-tooth stability, narrow occlusal table, maximum cusp height, and fossae depth with supplemental anatomy [24,25].

There is sufficient literature indicating the effect of the absence of posterior disocclusion and increased muscle activity [13,17,26,27]. Therefore, it is critical in every form of dentistry, whether in natural occlusion, restored occlusion, or bite splint therapy, to achieve the universal goal of posterior disocclusion.

Dawson [14] emphasized the disocclusion of posteriors in excursive movements irrespective of the amount. Except for Hobo et al. [17,21,22], the amount of disocclusion has been sparingly studied [28]. Hobo et al. [17,21,22] investigated molar disocclusion during eccentric movements in which the right and left condyles moved 3 mm in protrusive movement, and the nonworking condyle moved 3 mm in lateral movement. The amounts of disocclusion were 1.1 ± 0.6 mm during protrusive movement and 0.5 ± 0.3 mm on the working side, and 1.0 ± 0.6 mm on the nonworking side during lateral movement measured at the mesiobuccal cusp tip of the mandibular first molar. The amount of disocclusion obtained in the present study was greater than in studies done by Hobo et al.

In the present study, disocclusion was determined in excursive mandibular movements. The amount of disocclusion was recorded at the mesiobuccal cusp tip of the mandibular first molar.

The amount of disocclusion obtained by the direct technique for all three groups, i.e., protrusive, working, and nonworking, was greater than that obtained by the indirect technique. These results can be explained based on the mechanics of mandibular movements and the influence of cusp anatomy. When the mandible is protruded from maximum intercuspation, contact between the incisal edges of the mandibular anterior teeth and the lingual inclines of the anterior maxillary teeth results in an anteroinferior movement of the mandible. Thus, disocclusion increases as the subjects protrude from the 2 mm protrusion to the complete edge-to-edge position of the maxillary and mandibular central incisors.

Contraction of the inferior pterygoid muscle causes the nonworking condyle to move anteriorly and medially when the condyles are in the centric relation position. If the working side's inferior lateral pterygoid stays relaxed, the working condyle will remain in CR and rotate around the hinge axis [3]. This movement of the nonworking side in the inferior direction is responsible for the disocclusion of posterior teeth and the difference in indirect and direct record findings. While comparing the values obtained from both the groups, there was a statistically highly significant difference (p<0.01) seen for the disocclusion between all the groups except the left lateral working side (p˃0.05), with greater disocclusion seen with the direct technique.

On the working side, the shift of the rotating condyle during a lateral translation movement is determined by the morphology and ligamentous attachments of the TMJ undergoing rotation. The movement takes place within a 60-degree (or less) cone, with the apex at the rotational axis. Therefore, in addition to lateral movement, the rotating condyle can also move in the superior, inferior, anterior, or posterior direction. Furthermore, combinations of these, like laterosuperior or lateroinferior, can occur [3]. This complexity of movement of the working side condyle may be responsible for the statistically non-significant difference (p<0.05) between the direct and indirect techniques on the left working side.

A statistically significant positive and moderate correlation was seen between direct working and direct nonworking subgroups (r=0.497, p=0.009). A statistically significant positive and low correlation was noted between indirect working and indirect nonworking (r=0.325, p=0.041) and direct nonworking and direct protrusive subgroups (r=0.395, p=0.012).

A positive correlation indicates that when disocclusion on the working side increases, the same would be seen on the nonworking side for both the indirect and direct techniques. The direct technique showed a similar correlation in the protrusive and nonworking side disocclusion. The mandibular movement mechanics stated above may be responsible for these findings.

In the present study, silicone indices were made on an articulator, using the anterior teeth as a guide. The spherical end of the incisal pin moved 2 mm eccentrically on the anterior guide table. The amount of disocclusion during indirect protrusive, indirect nonworking, and indirect working averaged 1.22 ± 0.37 mm, 1.71 ± 0.61 mm, and 0.85 ± 0.33 mm, respectively.

The disocclusion obtained by the direct technique is greater than the indirect technique, and the values ensure the disocclusion of posterior teeth in function. It compensates for flatter returning pathways of the articulator's mandible and straight-line condylar pathways (14,17-19). The condyle follows a convex pathway, which will automatically cause a disocclusion of the posteriors intraorally when a minimum disocclusion of 1.2 mm, 0.8 mm, and 1.7 mm for protrusion, working, and nonworking side, respectively, is achieved on a straight-line articulator. Study limitations include a limited number of specimens, which need to be increased to obtain a more meaningful result.

## Conclusions

The following conclusions were drawn from this study: Direct records show higher values than indirect records. For both techniques, as the working side values increase, the nonworking side values increase too. Occlusal rehabilitation should incorporate a minimum disocclusion of 1.2 mm, 0.8 mm, and 1.7 mm for protrusion, working and nonworking sides, respectively, on the articulator to ensure disocclusion of posterior teeth intraorally. The quantification of disocclusion can provide the practitioner with a guideline of desirable disocclusion for a harmonious stomatognathic system.
